# Can Motion Graphic Animation About Snakes Improve Preschoolers’ Detection on Snakes? A Study of Inattentional Blindness

**DOI:** 10.3389/fpsyg.2020.609171

**Published:** 2021-01-15

**Authors:** Jie Fang, Jiangbo Hu, Fen Wang, Congcong Yan, Hui Zhang

**Affiliations:** ^1^Hangzhou College of Preschool Teacher Education, Zhejiang Normal University, Jinhua, China; ^2^Macquarie School of Education, Macquarie University, Sydney, NSW, Australia

**Keywords:** motion graphics, animation, preschool education, science education, inattentional blindness

## Abstract

This study created a motion graphic (MG) animation about the danger of snakes within a story telling structure, which is different from a traditional science animation that relies on explanatory language to explain the scientific concept. The effects of the two types of animations on children’s attentional perception on snakes were compared by an inattentional blindness (IB) task. Three groups of children undertook the IB task with one control group who did not watch the animation and the other two groups who watched the MG and the traditional styled animation, respectively in advance. The results showed that: (1) Children who watched the animation were significantly more likely to detect the unexpected snake images in the IB task than those who did not watch the animation; (2) Children who watched the MG animation showed a higher detection rate on the snake images than those who watched the traditional animation. The findings indicate that the intervention of animation would increase children’s attentional perception on the key concepts significantly. The MG animation has more impact than the traditional animation on children’s attentional perception on the key information. This study demonstrates that MG animation may have a significant value in promoting science education for young children that merits further explorations in depth.

## Introduction

Animation videos facilitate young children’s learning experiences with its entertaining nature attracting children’s interests and the vivid images matching their cognitive developmental stage that is sensitive to visualized information (Akpinar, 2014; Kim and Choi, 2018). Science animation, which integrates concrete audio-visual scientific information with a narrative structure, is an effective tool to attract young children’s attention and develop their scientific understanding and social skills (Roseberry et al., 2010; Liu and Chai, 2012). The produce of science animation for young children is a “synthesizing process” that mixes the elements of science, art, sounds, language, and social values (Wiana, 2018; Hapsari N. et al., 2019). Traditionally, explanatory language plays an important role in science animations for delivering the scientific knowledge. For example, Liu and Chai (2012) explored web-based interactive animation for primary school children’s safety education. The animation used in this study employed extensive language for the introduction about traffic rules and road safety that was combined with 2D and 3D pictures. They found that whilst 3D pictures seemed to be more inviting to children, both types boosted the children’s learning motivation and effectiveness. In this study, the animation which relies on the combination of language and 2D or 3D pictures for the presentation of the key information is named as traditional styled science animation. This type of animation has been strongly advocated and widely accepted for the promotion of young children’s scientific education.

Recently, motion graphic (MG) video animation, which focuses on giving movement to graphic design element, has been applied in science education. Compared with the traditional styled science animation, MG science animation possesses dynamic characteristics in emphasizing key information through moving images or symbols for capturing viewers’ notice and unpacking complex concepts (Ünal et al., 2010; Aksoy, 2012). Hapsari A. S. et al., 2019 applied MG animation to physics education in the primary school (Grade five) with a comparison of two groups of children learning the same lessons with and without the intervention of a MG animation for physics conceptual illustration. They found that the group who watched MG video animation achieved better learning results than the group undertaking the lesson in a traditional way without the support of the media. This study confirms the advantage of MG in unpacking the scientific concepts. However, traditional science animation may have the similar function in supporting children to gain more understanding in scientific concepts as well (Hatsidimitris, 2013). From the current literature, it is difficult to know the difference between the effect of MG and traditional science animation in terms of promoting children’s scientific understanding, though a few studies have shown that MG animation seems to be friendlier to young children (Hapsari A. S. et al., 2019). This study is an attempt to compare the effect of these two types of science animation on preschool children’s scientific conception, which has an implication for children’s learning animation creation.

To assess the effect of these two types of animation on children’s scientific perception, an inattentional blindness (IB) task is employed in the current study to exam children’s sensitivity to the key information delivered by the animation. The term of IB refers to a person’s “blindness” of an unexpected but visible stimulus when this person concentrates on another commission (Mack and Rock, 1998; Simons and Chabris, 1999). IB relates to people’s immediate information capturing process that is significantly affected by the personal differences, the attributes of the stimulus and the difficulty of the primary task (e.g., Most et al., 2005; Koivisto and Revonsuo, 2007; Kreitz et al., 2015). Research confirms that overt or covert meaning connection between a stimulus and an individual’s existing notion would increase the individual’s detection on the unexpected stimulus (Most et al., 2001; Rattan and Eberhardt, 2010). For example, in an IB task, the participants who had a preliminary experiences with the concept of African American had a higher detection rate on gorilla images than those who had primed on the concept of European American (Rattan and Eberhardt, 2010). This study demonstrates that the cultural related priming task may strengthen a meaning connection reflecting some people’s racial discrimination (African concepts are related to gorilla), which affected their detection rate of the unexpected stimuli. In regards with the current study, we use the science animation about snakes to convey the information that snakes are dangerous and always hidden in the bushland. The animation assists the construction of the meaning connection between the participating children’s notion of bushland-related snake images and life-threatening danger. Based on this principle, an IB task is designed with a bushland as the background and the snake image as an unexpected stimulus. It is conducted after the children watch the animation, through which the impact of the animation on children’s perception of snakes would be revealed.

The choice of snake for the topic of the animation and the IB task is based on the consideration that snakes represent threat-relevant stimuli that have been applied in studies for several decades regarding young children’s attentional perception (LoBue and DeLoache, 2008, LoBue and Deloache, 2011; Hoehl et al., 2017). Rapid detection of threat-relevant stimuli is critical for young children’s self-protection from danger. However, children do not always show attentional bias to threat-relevant stimuli (Lipp et al., 2004; Jackson and Calvillo, 2013). It is necessary to improve the education of children’s sensitivity to threat-relevant stimuli, yet little research has directly addressed this topic. Several empirical studies suggest that thematic education (e.g., traffic signs) based on highly structured courses or pictorial storybooks could increase children’s safety knowledge about the threatening hazards (Meir et al., 2015; Alonso et al., 2020). These studies assess the effectiveness of the intervention education program from the perspective of individual changes in relevant knowledge. The current study differs from the above research by focusing on children’s attentional perceptions on the danger of snakes that is beyond the relevant knowledge. Attentional perception directly relates to the hazard detection rate that is crucial for self-protection in practical situations, whilst knowledge underpins individual attentional perception, but may not directly associate with the hazard detection functions (Akio et al., 2015). The IB task of this study is to investigate the impact of the animation on children’s attentional bias on snake images rather than their knowledge about snakes. This research design provides a different view for evaluating the efficiency of science education.

To sum up, video animation is an effective media in science education that significantly contributes to children’s conceptual development. This study creates a narrative MG science animation with the topic about the danger of snakes in bushland, applying MG technology in a storytelling structure. It sets to examine whether this type of animation is more effective than the traditional animation in meaning making of the key concepts to children when both conveys the same information with the similar storytelling structure. An IB task is introduced to assess two groups of children’s attentional perception on snakes after watching the two types of videos. Meanwhile, we also set a control group who do not watch the animation before the IB task, but these children understand that snakes are dangerous from their previous learning experiences. This design is to explore the nature of children’s IB performance on snake detection, thus comparing children’s IB performance with and without the intervention of the “snake animation.”

Based on previous research, this study was carried out with the hypothesis that the children who watched the “snake animation” will have a higher “snake detection” rate in the IB task than those who did not watch the animation and the children who watched the MG animation about snakes will have a higher rate of “snake detection” than those who watched the traditional animation.

## Materials and Methods

### Participants

The participants were 65 preschoolers recruited from two preschools in Hangzhou, China. The selection standard was that children were physically and mentally healthy with normal or corrected-to-normal vision. To ensure the standard being met, a full attentional trial was designed in the process of data collection in which a stimulus showing on a screen should be detected when the participating children paid full attention to this task. Two children who could not detect the stimuli in this trial were removed from the data. Two children’s accuracy in the primary tasks was below 75%, which meant that they could not focus their attention on the primary tasks, were also excluded. Eventually a total of 61 children (31 boys) participated in this study. These participants were divided into three groups randomly. No Animation Group represented the children who had not watch the video animation before the IB task (21 children; *M*_*age*_ = 70.48 months, SD_*age*_ = 4.68 months; 11 boys). Traditional Animation Group indicated these children had watched a traditional science animation before the IB task (20 children; *M*_*age*_ = 72.21 months, SD_*age*_ = 2.32 months; 10 boys). MG Animation Group meant these children had watched the MG science animation before the IB task (20 children; *M*_*age*_ = 70.01 months, SD_*age*_ = 3.84 months; 10 boys). No significant difference showed on age (*F*(2,58) = 2.211 *p* = 0.119, and gender, χ^2^(2) = 0.031, *p* = 0.985) among the three groups.

The ethics of the study was approved by the Ethics Committee of Zhejiang Normal University (REC: ZSDR2019010). The information letter introducing the study object and the data collection process were sent to the parents and the educators of the two preschools. Consent forms were collected from the parents of the participating children.

### Materials

#### Animations

**FIGURE 1:**
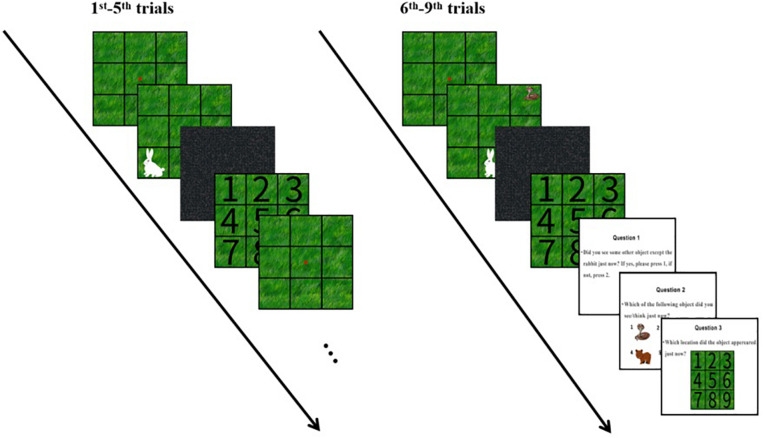
Flow diagram of the experiment. The 1st–5th trials were general trials in which only a rabbit was presented in the nine palace squares. The sixth to ninth trials were experimental trials in which an unexpected snake appeared in one of the nine palace squares. Three questions were asked immediately after the judgments.

**FIGURE 2:**
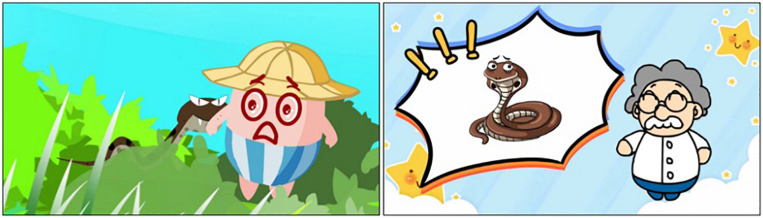
Some shots of the traditional science animation.

Two short educational animations, namely one MG and one traditional styled animation, were produced for this study. They shared one title “*Don’t Play in the Bush*” with the same storytelling structure in which a group of children played in the bush, resulting in a child being bitten by a snake hidden in the bush. However, the presentation styles of the two animations are different. The MG animation presented the story and used MG to emphasize the key information including; (a) the snake appeared and was surrounded by a long wavy grass; (b) the snake opened its’ mouth and hissed with the background of “thrilling” sounds. The traditional animation included the story which was similar to the MG animation, but followed with an explanation part in which a science doctor explained why the children would be bitten by the snakes. The science doctor’s explanation was combined with relevant pictures (e.g., snake pictures) as well. Both animations lasted 1 min. Several shots of the animations are shown in Figures 1, 2 and the animations could be uploaded in the Supplementary Material.

In addition, we invited 20 preschoolers to explore the difference of emotional status after watching these two animations. Four questions (interested, focused, scared, and hostile) relating to the current two animations were selected from the *Positive and Negative Affect Schedule*. The answers from the children showed no significant differences in the after-watching emotional status between these two animations, *t*(18) = 1.586, *p* = 0.130, cohen’s d = 0.709.

#### IB Task Program

The experimental program of the IB task is written by E-Prime 3.0 software. The stimulus consists three parts: (a) a big square in the center which is divided into nine palace squares and every square is covered by green grass (representing the bushland) (RGB: 34, 139, 34; visual angle: 9.6°); (b) a cartoon white rabbit (RGB: 255, 255, 255; visual angle: 2.5°) figure appears randomly in one of the nine palace squares (visual angle: 3.2°); (c) a red cross (RGB: 255, 0, 0; visual angle: 0.48°) always appears in the center of the screen; (d) an unexpected stimulus, a cartoon snake (RGB: 139, 71, 38; visual angle: 1.9°) figure in the current study, shows in one of the nine squares the same moment when the rabbit appears in another square in some trials.

### Procedure

**FIGURE 3:**
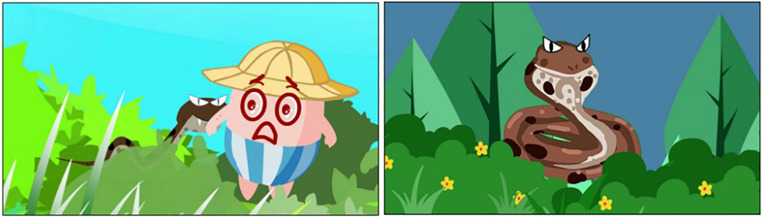
Some shots of the MG science animation.

As introduced previously, there were three groups of participating children in the experiment. The experiments of the IB task for each group were conducted individually. The IB tasks were presented on a laptop with a 14-inch monitor, a refresh rate of 60 Hz and a screen resolution of 1,024 × 768 pixels. The children were seated at approximately 60 cm from the screen. Two trained research assistants acted as the experimenters in the study. Figure 3 showed an illustration of the experiment. All the steps of the operation were completed by the participating children following the guidance of the experimenters. It took 5–8 min for the participating children to learn the instructions and complete the task.

There were nine trials in total. The first five trials were general trials and the last four were experimental trials. In the first five trials, the children were instructed to focus their attention on a red cross that helped them concentrate on the task and control their eye movement with an obvious focus, and then the rabbit would be presented in one of the squares and the children were required to point out where the rabbit appeared to the experimenter. In the sixth to ninth trials, an unexpected stimulus of a snake appeared in another square when the rabbit showed. The children would be asked three questions about the unexpected stimuli after they pointed out the location of the rabbit. After the questions, the participants would expect another stimulus’ appearance. Therefore, the seventh and eighth trials were defined as divided attentional trial 1 and divided attentional trial 2. The ninth trial was a full attentional trial. In the seventh trial, there was no specific cue of the unexpected stimuli though the children might hold the expectation, because the questions after the sixth trial were vague. For adults, most would detect the unexpected stimuli in this type of divided attentional trial (Mack and Rock, 1998). However, many young children cannot divide attention to another stimulus in a divided attentional trial (Zhang et al., 2019). We held the expectation that many young children would have a low detection rate on the snake image in the seventh trial. The eighth trial went further to provide the clear instruction that a snake image would appear when the rabbit showed. This trial was a completed divided attentional trial. These two divided attentional trials were designed for studying how the participating children would process the same stimuli differently under the two conditions. However, in the ninth trial, the children were instructed to pay a full attention to the additional stimuli without the need for completing the rabbit detection task. If the children could not detect the stimuli in this trial, they would be removed from the data because they were not appropriate for this task. These children either had difficulties to stay in line with the instructions or were unable to detect the stimuli shown in 1000 ms. At the end of the experiment, all participants were asked if they had been bitten by a snake, and no one said they had.

### Coding

In the sixth trial, if the children reported seeing another object and selected the right identity (the snake image) or the location of the object, or if they reported seeing no other object but they could select the right identity and location, these participants would be classified into a “NIB” (Non-Inattentional Blindness) category; otherwise, they were placed in the “IB” (Inattentional Blindness) category. The seventh and eighth trials were divided trials. For the seventh and eighth trial, if the children reported seeing another object and could select the right location or shape of the stimulus, or if they reported not seeing another object but could select the right location and shape of the stimulus, they would be classified into a “NDB1” (Non-Divided Blindness 1) category in the seventh trial and a “NDB2” (Non-Divided Blindness 2) category in the eighth trail; on the contrary, they were sorted into the “DB1” (Divided Blindness 1) category in the seventh trial and a “NDB2” (Non-Divided Blindness 2) category in the eighth trail. The ninth trial was a full attentional trial without the rabbit detection task. If the children reported seeing another object and could select the right location or shape of the stimulus, or if they reported not seeing another object but could select the right location and shape of the stimulus, they would be placed in a “NFB” (Non-Full Blindness) category; otherwise they were placed in a “FB” (Full Blindness) category and treated as invalid data.

## Results

The IB rates of different groups were shown in Table 1 and they were compared among different groups. The results showed that there was significant difference of IB rates in these three groups, χ^2^(2) = 23.624, *p* < 0.001. We compared the IB rates of every two groups and the results showed that the IB rate in No Animation Group was significantly higher than Traditional Animation Group, χ^2^(1) = 7.552, *p* = 0.006, and MG Animation Group, χ^2^(1) = 23.504, *p* < 0.001. Meanwhile, the IB rate in Traditional Animation Group was significantly higher than MG Animation Group, χ^2^(1) = 6.144, *p* = 0.013. A similar comparison of DB1 and DB2 rates found significant difference in the three groups, DB1, χ^2^(2) = 16.461, *p* < 0.001, DB2, χ^2^(2) = 5.343, *p* = 0.069. The DB1 and DB2 rates of No Animation Group were significantly higher than Traditional Animation Group, DB1, χ^2^(1) = 7.003, *p* = 0.008, DB2, χ^2^(1) = 1.888, *p* = 0.169, and MG Animation Group, DB1, χ^2^(1) = 12.596, *p* < 0.001, DB2, χ^2^(1) = 4.221, *p* = 0.040. No significant difference showed in DB1 and DB2 rates between Traditional Animation Group and MG Animation Group, DB1, χ^2^(1) = 2.105, *p* = 0.147, DB2, χ^2^(1) = 1.026, *p* = 0.311.

**TABLE 1:** IB and DB rates of the three groups.

Group	IB	DB1	DB2
No Animation Group	85.7%	47.6%	19.0%
Traditional Animation Group	45.0%	10.0%	5.0%
MG Animation Group	10.0%	0%	0%
χ^2^ test	χ^2^(2) = 23.624, *p* < 0.001	χ^2^(2) = 16.461, *p* < 0.001	χ^2^(2) = 5.343, *p* = 0.069

## Discussion

This study used an IB task to assess the effect of the science animation on children’s perception on the key information delivered by the animation. The results match our hypothesis that both of MG and traditional video animation about snakes can improve the participating preschooler’s detection on the unexpected snake image in the attentional task. The MG animation has a stronger impact on preschoolers’ attentional bias on snakes than the traditional animation.

The result of children’s higher detection rate in the inattentional and the divided attentional trials after watching the animation shows that the children’s conception on the snakes was strengthened by the animation. This is in a stark comparison to the control group children who acknowledged the fact that snakes are dangerous but were less sensitive to the snake image in the IB task. This finding is in line with previous studies indicating that children’s sensitivity to certain information would be increased if the information is delivered through visual and auditory channels (King et al., 2007; Witton et al., 1998), and this is one of the reasons for the explanation regarding animation’s significant role in promoting children’s conceptual development in science education (Roseberry et al., 2010; Liu and Chai, 2012). However, the previous studies explored the pedagogical functions of science animation from the perspectives of children’s improvement in conceptual knowledge or understanding (Ünal et al., 2010; Aksoy, 2012). This study has investigated the impact of animation on children’s attentional perception based on their performance in the relevant IB task, which is a new approach to assess the educational function of science animation, and this is especially the case for the information relating to children’s perception on threatening signs in safety education.

The comparison between MG video animation and traditional animation in terms of their effectiveness in promoting children’s attentional perception is rarely reported in literature. A few studies compared these two types of animation from the perspective of children’s preference and found that MG animation is friendlier to children than the traditional type (Hapsari A. S. et al., 2019). This study reveals that compared with the traditional animation which uses extensive language to explain the information, MG animation, which highlights key information with the amplified video and audio combination, may have a greater impact on young children’s conceptions on the relevant information. Comparatively speaking, MG is characterized in the simplified pictures and the flexible graphic movement, which has the advantage of presenting complex scientific concepts with vivid images. In this study, the MG animation video uses a series of simplified movable pictures of a snake to strengthen the key information of the danger of snakes, combining the movement of the grass, the motion of the text and the sound effect. This combination affects children’s concepts about snakes more effectively than the traditional animation, resulting in the children’s higher detection rate on snake stimuli in the IB task. Moreover, although there are no differences in the current subjective reports of emotional status, we still need to consider the effect of emotion when examining the mechanism of the effect of MG. There are some evidences that emotion plays an important role in improving visual salience (Ohman et al., 2001; Fugate et al., 2020). However, the ineffectiveness of emotion in the current study could be caused by the methods of emotional testing, and the objective indicators may reflect more information of emotion. The results implied that MG styled animation could be a desirable method for strengthening young children’s conception on certain information. The design of science animation for preschool children needs to consider the characteristics of information acquisition of young children.

A noticeable point regards the strength of MG integrating with storytelling to meet children’s interest as well as stressing the key information. Research suggests that storytelling is an efficient and practical method for nurturing young children’s understanding in abstract scientific concepts in science education (Hu et al., 2020). In fact, there are some attempts to employ storytelling MG animation for popular science programs, such as *Cells at Work*, that achieved impressive success in both educational and entertainment aspects (Kakihara et al., 2018). However, little research reports how this type of animation can be applied in young children’s science education. The study shows the potential for the application of “storytelling MG animation” in science education for young children that is privileged in attracting their learning attention and strengthening their conceptions on essential information that educators want to emphasize.

Whilst the value of MG is advocated, the pedagogical functions of traditional animation should not be ignored. According to the results that watching traditional animation could significantly improve the preschoolers’ detection on the unexpected and expected snake, we believe that the role of traditional animation is important for supporting children’s conceptual development. Although from the perspective of the children’s IB performance, the traditional animation is less effective than the MG animation, the traditional animation enhanced children’s sensitivity to the threat of snakes with more detailed relevant knowledge. The explanatory language in traditional animation can assist children to gain a better understanding in comprehensive scientific knowledge (Liu and Chai, 2012). If the study was designed to assess children’s knowledge about self-protection from the risk of snake attack, the traditional animation may show more strength than the MG animation. However, this study focuses on children’s sensitivity to snake images from the perspective of IB that was affected by the sensory and cognitive conspicuity of the stimulus (Hancock et al., 1990; Cassarino and Setti, 2016). Based on the dynamic forms of illustration, MG animation in this study has more strength than the traditional animation in the reinforcement of the sensory conspicuity relating to children’s conception on snakes, which may contribute to the effectiveness of children’s detection on the unexpected stimuli after watching the MG animation.

### Limitation and Future Research

This study is an early pioneer of using IB task to investigate the effect of different video animation on children’s perception. This study is an exploratory journey where we achieved exciting results and also found some limitations. Firstly, the control group did not access any information about snakes before the IB task, which could contribute to their much lower detection rate on the snake image than the children who watched the animation and were ready for the stimulus. If the children in the control group gained some clues from picture books or discussions about snakes to activate their perception of snakes, then the comparison between the control group and the two animation groups could be more solid for evaluating the effect of the intervention of animation on children’s perception of snakes. Secondly, the individual difference of the participants’ prior experience was not strictly controlled, for example, some children might have seen snakes previously or read some storybooks about snakes, which may also affect the results. Thirdly, it may be more convincing to record the objective report by polygraph to test children’s emotional status after watching these two animations, instead of using some subjective reports.

Future research addressing the above limitations would further clarify the pedagogical functions of different types of animation that has extensive implications for science animation creation for young children. Another avenue for the future research relates the application of MG animation for young children’s education in general. Whilst MG has been widely used in a variety of commercial and other educational fields that shows effectiveness in attention capture and notion enforcement to its viewers (Barnes, 2016, 2017), how MG can be applied in early childhood education is rarely reported. Given the potential educational functions shown in the current study, MG animation merits additional studies in the field of early childhood education.

## Data Availability Statement

The raw data supporting the conclusions of this article will be made available by the authors, without undue reservation.

## Ethics Statement

The studies involving human participants were reviewed and approved by Ethics Committee of Zhejiang Normal University (REC: ZSDR2019010). Written informed consent to participate in this study was provided by the participants’ legal guardian/next of kin.

## Author Contributions

JF and HZ designed the manuscript, collected the data, and wrote the manuscript. JH and CY wrote the manuscript. FW collected the data. All authors contributed to the article and approved the submitted version.

## Conflict of Interest

The authors declare that the research was conducted in the absence of any commercial or financial relationships that could be construed as a potential conflict of interest.
